# Cardiovascular disease behavioural risk factors in rural interventions: cross-sectional study

**DOI:** 10.1038/s41598-023-39451-5

**Published:** 2023-08-17

**Authors:** Adewale Samuel Akinosun, Sylvia Kamya, Jonathan Watt, William Johnston, Stephen J. Leslie, Mark Grindle

**Affiliations:** 1 Digital Health, Institute of Health Research and Innovation, University of the Highlands and Islands, Inverness, UK; 2 Cardiology Unit, Raigmore Hospital, NHS Highland, Inverness, Scotland, UK; 3 Insight Centre for Data Analytics, University College Dublin, Dublin, Ireland

**Keywords:** Cardiology, Diseases, Risk factors

## Abstract

This study aims to (1) assess the distribution of variables within the population and the prevalence of cardiovascular disease (CVD) behavioural risk factors in patients, (2) identify target risk factor(s) for behaviour modification intervention, and (3) develop an analytical model to define cluster(s) of risk factors which could help make any generic intervention more targeted to the local patient population. Study patients with at least one CVD behavioural risk factor living in a rural region of the Scottish Highlands. The study used the STROBE methodology for cross-sectional studies. Demographic and clinical data of patients (n = 2025) in NHS Highlands hospital were collected at the point of admission for PCI between 04.01.2016 and 31.12.2019. Collected data distributions were analysed by CVD behavioural risk factors for prevalence, associations, and direction of associations. Cluster definition was measured by assignment of a unit score each for the overall level of prevalence and significance of associations, and general logistics modelling for direction and significance of the risk. The mean (SD) age was 69.47(± 10.93) years [95% CI (68.99–69.94)]. The key risk factors were hyperlipidaemia, hypertension, and elevated body mass index (BMI). Approximately 40% of the population have multiple risk factor counts of two. Analytical measures revealed a population risk factor cluster with elevated BMI [77.5% (1570/2025)] that is mostly either hyperlipidaemic [9.43%, co-eff. (17), *P* = 0.007] or hypertensive [22.72%, co-eff. (17), *P* = 0.99] as key risk factor clusters. Carefully modelled analyses revealed clustered risk associated with elevated BMI. This information would support a strategy for targeting risk factor clusters in novel interventions to improve implementation efficiency. Exposure to and outcome of an elevated BMI is linked more to the population’s socio-economic outcomes rather than to regional rurality or urbanity.

## Introduction

### Background

The global burden of cardiovascular diseases (CVD) remains high^1^. In the UK, 7.4 million people live with CVD and approximately 460 deaths are recorded per day^2^. This burden accounts for a total annual healthcare cost of £800 million in Scotland^2^. The main therapeutic procedure for obstructive coronary artery disease is percutaneous coronary intervention (PCI) with over 500 procedures performed annually in the Scottish Highlands^3^. Despite the increase in annual counts of procedures and repeated sessions, there continue to be significant challenges to access especially in rural regions^4^.

Cigarette smoking, diabetes, obesity, and hypertension are independent risk factors for CVDs accounting for about 50% of its pathogenesis^1,2^. Treatment of these risk factors has been proven to reduce the risk of future cardiac events^5^. It has been predicted that mortality from CVD in the UK could be halved by small changes in cardiovascular disease risk factors—reducing smoking prevalence by 1% could lead to 2000 fewer CVD deaths per year, and a one percent reduction in population diastolic blood pressure could prevent around 1500 CVD deaths each year^2^. High-risk reduction strategies i.e. multiple risk factor treatment have also been suggested to have a substantial impact in CVD reduction^6^.

Following a cardiac event such as myocardial infarction or PCI for stable angina, cardiac rehabilitation programmes provide comprehensive rehabilitation in the form of education, exercise sessions, and psychological support. However, the impact of cardiac rehabilitation remains suboptimal due to (1) accessibility, (2) cardio rehabilitation class uptake and adherence, and (3) patient understanding of CVD^7^. In addition, recent studies have also indicated low rehab support acceptability and lack of completion of classes as major factors among people with lower health literacy^8,9^. A one-size-fits-all approach further weakens the efficacy of interventions due to the exclusion of patient experience in their design^10^. Digital technologies (such as software applications, the internet, and wearable sensors) in CVD risk factor modification have been embraced by some individuals as beneficial. They have the potential to overcome some of these challenges by delivering an alternative channel of educating, coaching and training out-patients^11^. However, digital technologies appear to be weak in reducing unhealthy behaviours linked with CVD risk factor modification when compared to cardiac rehab programmes^12^.

Several approaches have been used to help modify health behaviour among people with CVD risk factors. Common examples include health training and coaching sessions, health promotion and campaigns, and community-based participatory research^13^. Storytelling, which is also referred to as a narrative, has historically been used as a means of influencing public opinion and regulating human behaviour. More recently on a digital platform, it has been used for marketing and politics^14^. Lately, in the health sector, digital storytelling has been used for the modification of health behaviour in clinical trials and interventions especially in ‘remote rural’ and ‘deprived’ populations. Due to the ability to integrate patient experience in its design, digital storytelling has now been indexed as a major approach to different CVD-related risk factor modification.

The generalised approach used in the development of behavioural interventions is not fit for the purpose because it is not cost-efficient in the long term^15^. Therefore, novel (as well as existing) interventions such as digital storytelling in behavioural risk factor modification could use a methodological assessment or model of risk factors of interest in a specific population to identify which risk factor(s) to target. Such a model could inform a risk factor cluster target, which is geo-localized in its strategic pattern towards a patient-focused approach. This would create an effective targeted behavioural intervention application, in a more efficient and sustainable manner^15,16^. This study presents an analytical approach, which could be replicated in future intervention designs for different disease populations.

### Objectives

This study aims to (1) assess the distribution of variables within the population and the prevalence of CVD risk factors in patients, (2) identify target CVD risk factor(s) for behaviour modification intervention, and (3) develop an analytical model to define cluster(s) of risk factors which could help make any generic intervention more targeted to the local patient population.

## Methods

Defining population clusters by cause and effect (outcome) distribution could use various observational study designs such as cohort, case–control, or cross-sectional studies depending on the research focus. A cohort study design distinguishes between cause and effect by studying incidence, causes, and prognosis while a case–control study design seeks to identify possible predictors of an effect (usually a rare effect) by retrospectively comparing groups. However, a cross-sectional study design determines prevalence by simultaneously focusing on cause and effect in time^17^.

This study intends to model risk factors in clusters to determine how they interact around an outcome of interest, CVDs. This study chose a cross-sectional design because it is cost-efficient, not time-consuming, and does not need participant follow-up; to achieve the aims of the study^17^.

### Study design

This study was designed in line with Strengthening the Reporting of Observational studies in Epidemiology (STROBE) methodology for cross-sectional studies^18^. The population distribution was analysed for prevalence by gender, exposure to CVD risk factors, and number of risk factor counts. Statistical associations were tested between independent variables and risk factors, and the direction of association was determined.

This study does not require ethical approval or subject consent. However, approval for use of anonymised data was required. This was received from the office of the Caldicott Guardian, NHS Highland. The NHS Scotland guidance and regulation on the use of anonymised data of patients does not require recourse to patients on the use of data for the purpose of clinical research inputs meant for hospital benefits.

### Setting

Retrospective data from patients who had undergone PCI at Raigmore Hospital in Inverness, NHS Highland from 4th of January 2016 to 31st of December 2019 were included in the study. Eligible patient’s demographic and clinical data were collected at the point of admission for a PCI.

### Participants

Patients who have had at least one elective or emergency PCI.

### Variables

Demographic details include age, gender, geographic deprivation groups, economic deprivation ranks, family history of coronary artery disease, and risk factor counts; procedural, administrative, and clinical details such as body mass index, total cholesterol concentration, blood sugar concentration, blood pressure status and smoking status.

### Data source and measurements

Age at the time of the data collection was grouped into four ranges: below 40, 40–59, 60–79, and 80 and above years^19^. Geographic deprivation groups were derived from postcode data-match with the Scottish Index of Multiple Deprivation, SIMD 2019^20^. The SIMD 2019 defines geographical location postcodes in Scotland as six groups: ‘accessible rural’, ‘remote rural’, ‘accessible small towns’, ‘remote small towns’, ‘large urban areas’ or ‘other urban areas’—these groups were re-classified into ‘SIMD groups’ and expressed as ‘urban’, ‘accessible’ and ‘remote’. Economic deprivation ranks were derived from postcode data-match with the Scottish Index of Multiply Deprivation, SIMD 2020^20^. The SIMD 2020 defines geographical location postcodes in Scotland as economic rank 1 (most economically deprived data zone) to rank 6976 (least economically deprived data zone) and classified as ‘SMID ranking’ in quintiles from one to five. BMI ranges were defined using the WHO adults’ BMI classification: underweight (below 18.5), normal weight (18.5–24.9), pre-obesity (25.0–29.9), obesity class I (30.0–34.9), obesity class II (35.0–39.9), obesity class III (above 40)^21^. These were grouped into ‘low or normal weight’ (≤ 24.9) and ‘elevated BMI’ (≥ 25.0) to capture the preventive and corrective nature of intervention. Cholesterol concentration was defined using the BHF measurement and grouped ≤ 5 mmol/L as healthy and > 5 mmol/L as high^22^. Blood sugar and blood pressure were qualitatively defined from the original dataset.

Body mass index (BMI) was derived from patients’ weight and height data and measured in kg/m^2^. All dependent variables (BMI, total cholesterol, blood sugar concentration, and blood pressure) used the National Health Services, NHS Scotland measurement units^23^. These variables were grouped based on exposure as high cholesterol and healthy cholesterol (cholesterol concentration), diabetic and not diabetic (blood sugar concentration), hypertensive and not hypertensive (blood pressure), elevated BMI and not obese (BMI group), smoking and not smoking (smoking group). Units are available in appendices.

### Bias

The study data has a few repeat patient PCI visits resulting in point duplicates. This was noted and reported in the results section. The study data did not provide sufficient detail of collection for the cholesterol variable (for hyperlipidaemic exposure), resulting in missing data > 50%. Fitness analysis was conducted to measure the effect of this bias on concerned variable. Goodness of fit was tested to measure the representativeness of the data.

### Data analyses

The distribution of the population by gender was presented in tables. Tests for differences in means (Welch two sample t-tests) and equality of proportions (3-sample prop-tests) were conducted to check for variance between groups. The prevalence of each risk factor by exposure within the population was analysed by proportions. Risk factor counts proportions were reported for each risk factor within the population.

Missing data were checked (missing compare test) for fitness as missing completely at random (MCAR) to validate the nature of missingness in variables with > 10% missing data e.g. hyperlipidaemic variable, for exposure to cholesterol. Goodness of fit (Pearson’s Chi-squared test) was conducted to ascertain the representativeness of the data in the general population.

A test of association was performed (Pearson’s chi-square test) to detect if there was any significant relationship (1) across independent variables (population’s age, gender, deprivation groups, deprivation ranks, and risk factor counts) and CVD behavioural risk factor determinants (for all identified behavioural risk factors), and (2) within CVD behavioural risk factor determinants using a dependent variable of interest as a potential predictor based on initial association and prevalence scores. A unit score was assigned for overall level of prevalence and association significance across all CVD behavioural risk factor determinants. Unit scores were added to ascertain a preferential determinant of choice^16,19^.

Finally, the direction of risk in association was analysed for a preferred CVD risk factor determinant (general logistics modelling: odds ratio and co-efficient estimates) among notable predictors with significant association scores in order to inform a suggestive clustering for the purpose of targeting intervention design in the whole population.

Continuous data analysis was presented as means ± standard deviations (SD) while categorical data was presented in percentages. Data wrangling and analyses was done using the R Studio Version 1.3.1056 software^24^. All tests were two-tailed with level of significance set at *P* < 0.05, and 95% Confidence Interval (CI).

### Role of funding source

The sponsors, as acknowledged in this text were not involved in study design; the collection, analysis and interpretation of data, the writing of the report or the decision to submit this paper for publication.

## Results

### Participants

In total, there were 2,025 patient data with a mean (SD) age 69.47 (± 10.93) years [95% CI, 68.99–69.94]. The women were older compared to men, mean age 71.14(± 11.1) to 68.91(± 10.8) years (*P* = 0.0001). Detailed characteristics (duplicates and missing data) of participants are described in Table 1.

**Table 1:** The NHS Highlands CVD PCI population distributions by gender, 2016–2019.

	Total (%)	Female (%)	Male (%)	*P* value
	n = 2025^1^ (100)	n = 510 (25.2)	n = 1515 (74.8)	.
Age, µ (± SD) years	69.5 (± 10.9)	71.1 (± 11.1)	68.9 (± 10.8)	0.0001
Below 40	16 (0.7)	5(0.2)	11 (0.5)	0.0001
40–59	426 (21.1)	80 (4.0)	346 (17.1)	0.0001
60–79	1245 (61.5)	318 (15.7)	927 (45.8)	0.0001
80 above	337 (16.7)	107 (5.3)	230 (11.4)	0.0001
Family history, CAD	869 (42.9)	213 (41.8)	656 (43.3)	0.76
Diabetes	426 (21.0)	97 (19.0)	329 (21.7)	0.22
Dietary	378 (18.7)	83 (16.3)	295 (19.5)	.
Insulin	48 (2.4)	14 (2.7)	34 (2.2)	.
Hyperlipidaemia^2^	543 (26.9)	166 (32.5)	377 (24.9)	0.002
Hypertension	1099 (54.3)	286 (56.1)	813 (53.7)	0.37
Obesity (BMI status)	.	.	.	0.0001
Elevated BMI	1570 (77.5)	356 (69.8)	1214 (80.1)	.
Low/normal weight	453 (22.4)	153 (30.0)	300 (19.8)	.
Smoking status	.	.	.	0.001
Current smoker	458 (22.7)	139 (27.3)	319 (21.1)	.
Ex-smoker	815 (40.2)	169 (33.1)	646 (42.6)	.
Never smoked	749 (37.0)	201 (39.4)	548 (36.2)	.
SIMD groups^3^	.	.	.	0.29
Accessible	244 (12.0)	69 (13.5)	175 (11.6)	.
Urban	518 (25.6)	116 (22.7)	402 (26.5)	.
Remote	1140 (56.3)	295 (57.8)	845 (55.8)	.
SIMD ranking^4^	.	.	.	0.17
1	170 (8.4)	45 (8.8)	125 (8.3)	.
2	316 (15.6)	96 (18.8)	220 (14.5)	.
3	690 (34.1)	172 (33.7)	518 (34.2)	.
4	591 (29.3)	136 (2.6)	455 (30.0)	.
5	173 (8.6)	41 (8.0)	132 (8.7)	.
Risk factor count	.	.	.	0.15
0	96 (4.7)	31 (6.1)	65 (4.3)	.
1	504 (24.9)	122 (23.9)	382 (25.2)	.
2	801 (39.6)	188 (36.9)	613 (40.5)	.
3	512 (25.3)	133 (26.1)	379 (25.0)	.
4	106 (5.2)	33 (6.5)	73 (4.8)	.
5	6 (0.3)	3 (0.6)	3 (0.2)	.

SIMD, Scottish index of multiple deprivation; CAD, coronary artery disease.

^1^Duplicates represent 345 counts and makes up to about 17% of the population dataset.

^2^Missing data represents 889 counts and makes up to 44% of the population.

^3^Missing data represents 123 counts and makes up to 6% of the population.

^4^Ranking is in quintiles. Missing data represents 85 counts and makes up to 4.2% of the population.

### Data description

Table 1 presents the population demographic and clinical data distribution by gender with *P* values (t-test and prop-test) for difference in means and equality of proportions. The hyperlipidaemia (cholesterol) variable was marred with missing values by 44% (892 of 2025). Test for fitness (in comparison to independent variables, representative of the population, such as ‘age’ and ‘distance from hospital’) shows that missing data was not MCAR at *P* < 0.05. Additional fitness check (using the gender variable, which is also representative of the whole population) shows that missing value were not significantly different from observed values for proportions in both male (842 (55.6%), 673 (44.4%)) and female (294 (57.6%), 216 (42.4%)) populations (*P* = 0.45).

### Prevalence of risk factor exposures by independent variables

Table 2 presents the distribution of variables by proportion for CVD risk factor exposures.

**Table 2:** The prevalence of CVD risk factor exposures by independent variables in NHS Highlands CVD PCI population, 2016–2019.

	Blood sugar concentration (%)		Blood pressure (%)		BMI groups (%)		Cholesterol concentration (%)		Smoking groups (%)	
	Diabetic	Not diabetic	Hyper-tensive	Not hyper-tensive	Elevated BMI	Low/normal weight	High	Healthy	Smoking	Not smoking
	21.1	78.9	54.3	45.7	77.5	22.5	26.9	29.1	22.7	77.3
Gender	.	.	.	.	.	.	.	.	.	.
Female	4.8	20.4	14.1	11.1	17.6	7.5	8.2	6.3	6.9	18.3
Male	16.2	58.6	40.2	34.7	60.0	14.8	18.6	22.9	15.8	58.9
Family history of CAD	.	.	.	.	.	.	.	.	.	.
No	13.1	43.5	30.5	26.1	42.7	13.8	13.0	15.6	12.4	44.1
Yes	7.9	35.1	23.5	19.5	34.5	8.4	13.6	13.4	10.0	32.9
SIMD groups	.	.	.	.	.	.	.	.	.	.
Urban	5.5	20.1	13.6	12.0	19.4	6.2	6.9	7.6	6.6	19.0
Accessible	2.4	9.7	6.2	5.9	9.7	2.3	3.1	3.8	2.5	9.5
Remote	12.1	44.2	31.2	25.1	43.7	12.6	15.2	16.6	12.1	24.2
SIMD ranking	.	.	.	.	.	.	.	.	.	.
1	1.6	7.2	4.7	4.1	6.8	2.0	1.8	2.2	3.9	4.9
2	4.1	12.2	9.0	7.3	12.3	4.0	4.3	4.0	5.5	10.8
3	6.8	28.8	19.5	16.1	27.6	7.9	9.6	10.8	7.5	28.0
4	6.8	23.7	16.6	13.9	23.8	6.6	8.5	10.3	4.9	25.5
5	1.8	7.2	4.5	4.4	7.1	1.9	2.5	2.6	1.0	7.9
Risk factor count	.	.	.	.	.	.	.	.	.	.
0	0	4.7	0	4.7	0	4.7	0	2.0	0	4.7
1	1.1	23.8	4.4	20.4	14.6	10.3	1.8	7.6	2.9	21.9
2	4.6	35.0	22.7	16.8	33.5	6.0	9.4	12.3	8.9	30.6
3	11.4	13.9	21.8	3.5	24.0	1.3	11.3	6.6	7.4	17.9
4	3.6	1.6	5.0	0.2	5.2	0	4.0	0.6	3.1	2.1
5	0.3	0	0.3	0	0.3	0	0.3	0	0.3	0

### Prevalence of CVD behavioural risk factors by risk factor counts.

**Figure 1:**
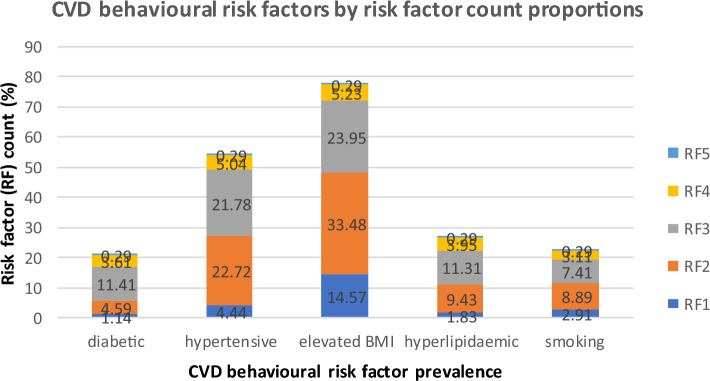
Prevalence of CVD behavioural risk factors by risk factor counts in the NHS Highlands CVD PCI population, 2016–2019.

Figure 1 presents the prevalence of CVD behavioural risk factors by risk factor counts (multiple exposures within the population).

### Test for association

Table 3 presents the association between independent variables and CVD behavioural risk factor determinants. Association scores and prediction scores are indicated by the counts of significant associations and levels of predictions, respectively.

**Table 3:** Tests and scores of associations between independent variables and CVD risk factor determinants in the NHS Highlands CVD PCI population, 2016–2019.

	Blood sugar concentration	Cholesterol concentration	Blood pressure	BMI groups	Smoking groups	Prediction score^3^
age groups	< 0.0001	< 0.0001	< 0.0001	< 0.0001	< 0.0001	5
gender	0.21	< 0.0001	0.36	< 0.0001	< 0.0001	3
family history, CAD	< 0.0001	< 0.0001	0.87	< 0.0001	< 0.0001	4
SIMD groups	0.85	0.23	0.62	< 0.0001	0.35	1
SIMD ranking	0.14	< 0.0001	0.86	0.87	< 0.0001	2
risk factor count	< 0.0001	< 0.0001	< 0.0001	< 0.0001	< 0.0001	5
Association score^1^	3	5	2	5	5	.
Risk prevalence score^2^	1	3	4	5	2	.
Preference score^1+2^	4	8	6	10	7	.
BMI groups	< 0.0001	0.01	< 0.0001	.	< 0.0001	

^1^Significant association counts, 1 (no significant association count, 0).

^2^Prevalence range, 1–5 (from Table 1 and Fig. 1: least to most prevalent).

^3^Counts of associations with dependent variables, 1 (no count, 0).

^1+^^2^Value of association score plus value of prevalence score.

### Test for direction of associations

Table 4 presents a generalised linear model odd ratio and coefficient estimates (where odd ratios were over-estimated) with their respective confidence intervals and p values for exposure to obesity using ‘age’, ‘gender’, ‘family history CAD’, ‘risk factor count’, ‘SIMD groups’ (deprivation groups), and other risk factor determinants as predictors.

**Table 4:** Summary of generalised linear model to determine level and direction of association in determinants for elevated BMI, showing odd ratios (OR) and co-efficient estimates (Co-eff.) in the in NHS Highlands CVD PCI population, 2016–2019.

	OR (conf. int.)	Co-eff. (P-value)
Age	1 [0.96, 1.23]	.
Gender (Male)	3 [0.12, 75.31]	.
Risk factor count	1 [0.24, 4.62]	.
Family history, CAD (No)	.	− 2 (0.99)
Family history, CAD (Yes)	.	3 (1)
Deprivation groups (Accessible)	.	1 (0.63)
Deprivation groups (Urban)	.	20 (0.99)
Deprivation groups (Remote)	.	20 (0.99)
Blood sugar concentration (Not diabetic)	.	1.3 (0.35)
Cholesterol concentration	.	17 (0.007)
Blood pressure (Not hypertensive)	.	17 (0.99)
Smoking groups (Smoking)	.	5 (0.006)
Smoking groups (Non-smoking)	.	23 (0.99)

## Discussion

### Key results

This study presents data analyses of CVD risk factors for patients living in a remote region who have undergone PCI over a period of four years. Data duplicates representing about 17% of the population revealed the annual burden of repeated procedures and extent of behaviour change challenge. Results show that elevated BMI (pre-obese and obese status) is the most prevalent CVD risk factor in the population with a significant difference in proportions in both gender (*P* < 0.0001), followed by hypertension (*P* = 0.37) and hyperlipidaemia (*P* < 0.002), with which further analysis shows existence of highest and multiple attributable risk within the population^25^.

A carefully modelled analyses by assessing overall prevalence, association significance, and direction of risk reveal a population with elevated BMI which is either hyperlipidaemic or hypertensive as clusters of interest for health behaviour change intervention.

### Limitation

This study dataset contains some missing data in the ‘cholesterol concentration’ variable, which had a significant count of missing values beyond the 10% theoretically benchmarked for the study. Secondly, the whole dataset is from a single centre and only looked at those who had a PCI intervention, which was not fully representative of the whole exposed population at risk. The bias in these limitations were either provided for or noted with their effects in the study.

Thirdly, the SIMD standard captures data based on area postcode. It is worthy of note that a pocket of individuals might deviate significantly from the general population socio-economic characteristics. However, from a public health perspective, an intervention might be desirable and designed based on the consideration of data from a larger percentage of a population.

Lastly, in addition to these limitations, survivor bias was also noticed. The only group of people that could be included in this study data were individuals who had survived a cardiac event. There is a chance that those who could have benefited from an intervention had died of stroke or myocardial infarction or decided not to go to the hospital after a first cardiac event.

### Interpretation

#### Confounders and determinants

In this study, age and risk factor count variables were significantly associated with all CVD risk factors. Though supported by clinical reports^26^, further tests indicating the level and direction of association were conducted. They showed that changes in these determinants did not have any effect (OR = 1) on exposure to obesity as a major and dominant CVD risk factor and may therefore be considered confounders within the population—all patients were equally exposed to being obese irrespective of age or number of risk factor counts. This feat agrees with study findings by Ng et al.^27^. The exposure effect (OR = 1) of risk factor count is validated in that elevated BMI is a dominant risk factor within the population. This, when adjusted for, suggests highest multiple risk association level for obesity compared to other CVD risk factors, an observation similar to study by Mora et al.^25^.

Association scores (Table 3) showed that gender and SMID group variables merit some discussion. The former as the sole associate with BMI groups and the latter, BMI groups and smoking group, a finding similar to study by Damen et al.^28^. Lastly, though with lower population proportion, the female gender has higher chance (OR = 3) of being obese compared to the male. This finding validates gender as a determinant of exposure to obesity as also indicated in clinical reports by NHS Scotland^23^.

#### Rurality and obesity

In comparing rurality and remoteness, the study results showed that living in a rural area does not completely explain being obese^13^. The chances (Co-eff = 20, *P* = 0.99) of being obese in the study population are high and equal for both rural and urban groups—this may be due to inaccessibility to health facilities. This suggests that rural dwellers may not be regionally deprived when compared with their remote urban counterparts within a geographically remote population as also reflected in the study done by Teckle et al.^29^. However, this finding could not affirm socio-economic status for the study population as the SIMD ranking (a socio-economic variable) did not indicate a significant level of association with all the CVD risk factor determinants except in cholesterol concentration and the smoking group variables. This observation is similar to the Scottish Government report on Tobacco intervention^30^. It is worthwhile to note that a unit change to geographical accessibility did not have any effect on the chance of being obese. This suggests and affirms that exposure to and outcome of an elevated BMI is linked more to social-economic outcomes rather than to rurality or urbanity as supported in previous studies^20,31^.

#### Family history of CAD and obesity

Results showed that having a family history of CAD increases the chance (OR = 3) of being obese and not having a family history of CAD decreases chance (OR = -2) of being obese, an observation similar to studies done by Jin et al.^32^.

#### Diabetes and obesity

For this study, the chance of being diabetic increases for individuals with obesity compared to the individuals without obesity, an observation supported in the clinical report by Diabetes, UK^26^. This association strength is 1.3 times as likely in obese individuals compared to their non-obese counterparts. This is not surprising as obesity is causally linked to diabetes.

#### Hypertension and obesity

In the blood pressure variable, a unit change in obesity increases the chance (co-eff = 17, *P* = 0.99) of being hypertensive. To affirm association strength, obese individuals are seventeen times as likely to be hypertensive compared to their non-obese counterparts. This observation on association strength further indicates a stronger level of association between hypertension and elevated BMI within this population compared to any other CVD risk factor. This, therefore, suggests the need for imminent intervention within observed cluster, a suggestion similar to study by Cesana et al.^33^.

#### Hyperlipidaemia and obesity

Though with *missing at random* data within the population, the significant association in the cholesterol concentration variable coupled with association strength for hyperlipidaemia is noticed with the elevated BMI group. This makes hyperlipidaemia and obesity a cluster risk factor of choice as also suggested by Iliodromiti et al.^34^.

#### Smoking and obesity

A previous report (2020) on tobacco suggested that the Scottish Government’s intervention(s) already in place to reduce smoking within the study population seems to be increasingly effective^30^. This suggestion is validated in that the ex-smoker individuals within the non-smoking population has the highest prevalence within the smoking group. This validation appears to be responsible for the higher chance (co-eff. = 23, *P* = 0.99) of being obese within the non-smoking population compared to the chance (co-eff. = 5, *P* = 0.006) of being obese in the smoking population as validated in the study by Ginawi et al.^35^. However, quitting smoking may be responsible for diminishing marginal effect on BMI thus reducing exposure to obesity as also reflected in study by Courtemanche et al.^36^.

### Generalizability

The study dataset is geographically localized—while its model may be considerably replicable for advisory use in public health behavioural risk factor interventions, the data outcomes may not be directly representative of intervention application in regions of the world with different CVD risk factor cluster profile. Studies have shown that CVD risk factor cluster profiles are region-specific^37,38^. The analytical model in this study could therefore be used to make any generic intervention more targeted to specific local populations.

In addition to this, it is worthy of note that in addition to smoking, behavioural risk factors such as unhealthy diet, alcohol consumption, and physical inactivity are important, and they play significant role and contribution toward exposure to clinical risk factors and CVDs. We suggest that future studies focused on risk factors in rural areas are conducted to provide more knowledge and insight.

## Conclusion

Carefully modelled analysis measures revealed clustered population of CVD risk factors with elevated BMI. It is therefore concluded that

1. The knowledge of population cluster structure could strategically and substantially inform cardiac rehabilitation intervention targets by improving implementation efficiency and effectiveness thereby further contributing to reduction in the burden of repeated procedures on existing clinical interventions.
2. Exposure to and outcome of an elevated BMI is linked more to the population’s socio-economic outcomes rather than to regional rurality or urbanity.

## Data Availability

The use of data was approved by the office of the Caldicott Guardian, NHS Highland. Data sharing is limited to the NHS Highland and University of the Highlands and Islands.
